# COVID-19 in City Council Civil Servants, 1 March 2020–31 January 2023: Risk of Infection, Reinfection, Vaccine Effectiveness and the Impact of Heterologous Triple Vaccination

**DOI:** 10.3390/vaccines12030254

**Published:** 2024-02-28

**Authors:** Luca Cegolon, Francesca Larese Filon

**Affiliations:** 1 Department of Medical, Surgical & Health Sciences, University of Trieste, 34128 Trieste, Italy; larese@units.it; 2 Occupational Medicine Unit, University Health Agency Giuliano-Isontina (ASUGI), 34148 Trieste, Italy

**Keywords:** COVID-19, SARS-CoV-2, primary infections, reinfection, vaccine effectiveness, civil servants, heterolous vaccination, vaccine effectivenss, occupational risk, healthcare workers

## Abstract

**Background**: The risk of COVID-19 increases in any occupation entailing intense social interactions. This study aimed to investigate the impact of COVID-19 among civil servants of Trieste city council (northeastern Italy) over the entire pandemic. **Methods**: The crude incidence rate of COVID-19 was estimated from 1 March 2020 to 31 January 2023 by explanatory factors, expressing the estimate as COVID-19 events x 10,000 person-days (P-d) at risk. A multivariable Cox proportional hazard regression model was fitted to examine the risk of primary COVID-19 infection and reinfections, reporting adjusted hazard ratios (aHR) with 95% confidence interval (95% CI). **Results**: The cohort of Trieste city council was mainly composed of administrative clerks (48.5%), nursery teachers (33%), technicians (9.9%) and local police officers (8.5%). Between 1 March 2020 and 31 January 2023, 1444 (62.4%) employees tested positive for SARS-CoV-2 at least once and 18.1% (=262/1444) at least twice. By the end of this study, 55% (N = 1272) of employees had received at least three doses of COVID-19 vaccine, whereas 19.7% (N = 457) remained unvaccinated. At multiple Cox regression analysis, the adjusted risk of primary COVID-19 events during the entire study period increased in employees aged 40–49 years (aHR = 1.65; 95% CI: 1.01; 2.71), females (aHR = 1.28; 95%CI: 1.12; 1.45), local police officers (aHR = 1.82; 95%CI: 1.50; 2.22) and nursery teachers (aHR = 1.27; 95%CI: 1.13; 1.43). However, whilst the risk of primary infections in police officers increased already during the Alpha transmission period (aHR = 6.82; 95%CI: 4.48; 10.40), progressively reducing across subsequent variants, for nursery teachers, it increased during the Delta wave (aHR = 2.42; 1.70; 3.44), reducing with Omicron (aHR = 1.23; 95%CI: 1.07; 1.40). Compared to unvaccinated colleagues, during the entire study period the risk of primary infections was significantly lower in employees immunized with three (aHR = 0.42; 95%CI: 0.36; 0.47) or four (aHR = 0.30; 95%CI: 0.23; 0.40) doses of COVID-19 vaccine, for a vaccine effectiveness (VE) of 58% and 70%, respectively. The protective effect of vaccination against primary infections was confirmed in the sub-group analysis by main pandemic waves, for a VE of 75% for one dose against 99% for two doses during the Alpha transmission period, slightly reducing to 59% and 70% in Delta time, respectively. During the Omicron wave, the risk of primary SARS-CoV-2 infections diminished significantly with three (aHR = 0.42; 95%CI: 0.36; 0.49) or four vaccine doses (aHR = 0.09; 95%CI: 0.05; 0.16), for a VE of 58% and 91%, respectively. Moreover, the risk of primary SARS-CoV-2 reinfections during the entire study period reduced with one (aHR = 0.47; 95%CI: 0.27; 0.82), two (aHR = 0.42; 95%CI: 0.30; 0.58), three (aHR = 0.32; 95%CI: 0.24; 0.44) or four vaccine doses (aHR = 0.14; 95%CI: 0.05; 0.46), for a VE of 53%, 58%, 68% and 86% against reinfections, respectively. No significant difference in VE was associated with heterologous versus homologous triple vaccination, both against primary infections or reinfections. **Conclusions**: Primary SARS-CoV-2 infections were more likely among nursery teachers and local police officers. The risk of both primary infections and reinfections reduced with higher number of doses of COVID-19 vaccine, regardless of the pandemic wave. Since city council civil servants were swab tested on demand or for contact tracing, the estimation of COVID-19 risk and VE largely missed aymptomatic SARS-CoV-2 infections. On the one hand, the present study confirmed the protective effect of COVID-19 vaccination against symptomatic SARS-CoV-2 infections; on the other hand, it highlighted not only the importance of continuous booster doses to keep up the humoral immunity over time but also the importance of updated vaccine formulations to prevent and control the spread of a highly mutable virus. Moreover, the protective effect of the first two doses against reinfections confirmed the efficacy of hybrid immunity during Omicron time.

## 1. Background

Although healthcare workers (HCWs) have been extensively investigated during the pandemic, any indoor occupational activity with intense social interactions inherently enhanced the risk of COVID-19 [1,2,3,4,5]. Remote work or occupations with minimal contact with the public or customers were, in fact, reportedly associated with the lowest risks of SARS-CoV-2 infection [6].

Different job exposure matrices (JEMs) were developed to estimate the risk of COVID-19 in relation to occupation. According to a Mat-O-Covid JEM established in France, the proportion of COVID-19 infections attributable to occupational exposure among 18,999 workers with 389 different job tasks was estimated to vary by 20–40% [7].

A COVID-19 job exposure matrix (COVID-19-JEM) was developed by experts from Denmark, the Netherlands and the UK considering the biological risk (number of contacts, nature of contacts, contaminated worksites, location of the worksite), risk reduction measures (social distancing and use of face mask) and degree of instability of occupation (proportion of workers with income insecurity and proportion of migrant workers) [8]. A clear exposure–response relationship was noted in the early phase of the pandemic, with increasing scores related to number of contacts, nature of contacts and social distancing [8].

According to a Job at Risk Index (JARI) applied in Belgium during the first pandemic year, all job sectors featured by interpersonal proximity without systematic SARS-CoV-2 exposure (education, law enforcement, fitness, beauty, retail, musicians/actors, restaurants, bars and transportation) exhibited the same incidence rate of COVID-19 infections [9]. 

In Italy, a methodological approach was implemented to classify the biological risk of SARS-CoV-2 infection in each economic sector as low, medium–low, medium–high or high depending on three parameters: exposure probability; proximity index; aggregation factor. According to compensation claims submitted to the Italian National Institute for Compesation of Work Related Injuries and Occupational Diseases (INAIL), a substantial proportion of COVID-19 infections (19.4%) were deemed work-related. Although healthcare and social services accounted for the large majority (71.6%) of occupational COVID-19 events in the latter Italian study, 10.4% of cases were still associated with civil (10.4%) or administrative (4.1%) services [10]. 

In view of the above, considering the dearth of epidemiological evidence on civil servants, this study investigated the risk of COVID-19 among employees of Trieste city council (northeastern Italy) from the beginning of the pandemic (1 March 2020) to 31 January 2023. 

In Italy, civil servants are an invaluable target population for assessing vaccine effectiveness (VE) of non-RNA vaccines and heterologous triple vaccination, since a large proportion of these workers were offered adenoviral vector DNA vaccines for the first two doses of COVID-19 vaccines.

## 2. Methods

### 2.1. Ethical Considerations

This study was approved by the regional ethics committee (CEUR) of Friuli-Venezia Giulia region (Reg N.H32/2021).

In compliance with Italian legislation on privacy law, informed consent from study participants was waived, since patients’ data were routinely collected for healthcare purposes and were managed anonymously within the framework of an approved study protocol. This study followed the Strengthening of Observational Studies in Epidemiology (STROBE) reporting guidelines.

### 2.2. Study Endpoint

The incidence of COVID-19 was investigated among 2314 employees of Trieste city council (northeastern Italy) from the start of the pandemic in Italy (1 March 2020) to 31 January 2023.

In compliance with the surveillance system of the local public health department of the University Health Agency Giuliano-Isontina (ASUGI), city council employees were swab tested for contact tracing or on demand, in case of symptoms consistent with COVID-19. No systematic mandatory testing schedule was therefore enforced in this occupational group.

Information on sex, age, occupation, date of positive swab test performed and date of any COVID-19 vaccine dose received was available for the analysis.

### 2.3. Statistical Analysis

Age (in years) was presented as mean and standard deviation (SD) and compared by different categories using a t-test. Categorical variables (sex, occupation, COVID-19 vaccination status before SARS-CoV-2 infection) were reported as numbers and percentages and compared using a chi-square test. 

The crude incidence rates of SARS-CoV-2 infections were calculated as number of events x 10,000 person-day (P-d) at risk, with 95% confidence interval (95%CI) by explanatory factor (age, sex, occupation and COVID-19 vaccination status) across the entire study period and by main pandemic waves (Wuhan, Alpha, Delta, Omicron). The risk of infection in relation to COVID-19 vaccination was estimated by time since last dose received (14+ days since first dose or 7+ days after 2+ doses). 

A multivariable Cox proportional hazard regression model adjusted for age, sex, occupation and COVID-19 vaccination status was fitted to investigate the risk of primary SARS-CoV-2 infections over the entire study period by main COVID-19 waves (Wuhan, Alpha, Delta, Omicron) and by heterologous versus homologous triple vaccination. Likewise, the risk of primary SARS-CoV-2 reinfections was estimated among workers previously SARS-CoV-2 infected once, estimating also the efficacy of heterologous verus homologous immunization against reinfecitons 

Results were reported as adjusted hazard ratios (aHR) with 95% CI.

VE [(=1 − aHR) × 100] was estimated against primary SARS-CoV-2 infections (in the entire cohort, by COVID-19 wave and by heterologous vs. homologous triple vaccination) and reinfections (in the entire cohort and by heterologous vs. homologous triple vaccination). 

Missing values were excluded, and a complete case analysis was performed.

The analysis was carried out with STATA 16.0 software (StataCorp LLC, College Station, TX, USA).

## 3. Results

Table 1 displays the distribution of COVID-19 cases among 2314 employees of Trieste city council from 1 March 2020 to 31 January 2023 (35 months), by explanatory factors. The median age of study subjects was 53 (IQR: 46;58) years, and the majority (70.4%) were females. The cohort of civil servants was mainly composed of administrative clerks (48.5%), followed by nursery teachers (33%), technicians (9.9%) and local police officers (8.5%). 

During the entire study period, the cumulative crude incidence of COVID-19 was 62.4% (=1444/2314) for primary infections and 18.1% (=262/1444) for primary reinfections. Ten workers were reinfected twice and two were reinfected three times (Table 2). Although no clinical information on individual COVID-19 symptoms was available, only 20 (0.01%) workers were hospitalized for COVID-19: 17 were unvaccinated (6 were hospitalized before the vaccine era); 1 had been immunized with two doses; 2 had been immunized with three doses. Only one worker, previously vaccinated with a triple dose, died. The remaining workers developed mild–moderate COVID-19 not requiring admission to hospital.

The rate of primary infections was significantly higher in females (64.6% = 1052/1629) than in males (57.2% = 392/685). The highest rate of primary COVID-19 infections (66.5% = 395/594) and reinfections (22.5% = 89/395) was observed in the 40–49 years age group. The frequency of primary infections was higher in administrative clerks (46.5% = 672/1,444) or nursery teachers (35.4%=511/1,444). Likewise, the frequency of primary reinfections was higher in nursery teachers (42.8%=112/262) or administrative clerks (40.1%=105/262). By contrast, the rates of primary SARS-CoV-2 infections (8.2%=135/198) and reinfections (23.0%=31/135) were higher among police officers followed by nurdery teachers (66.9% = 511/764 for primary infections vs. 21.9%=112/511 for reinfections) (Table 1).

**Figure 1:**
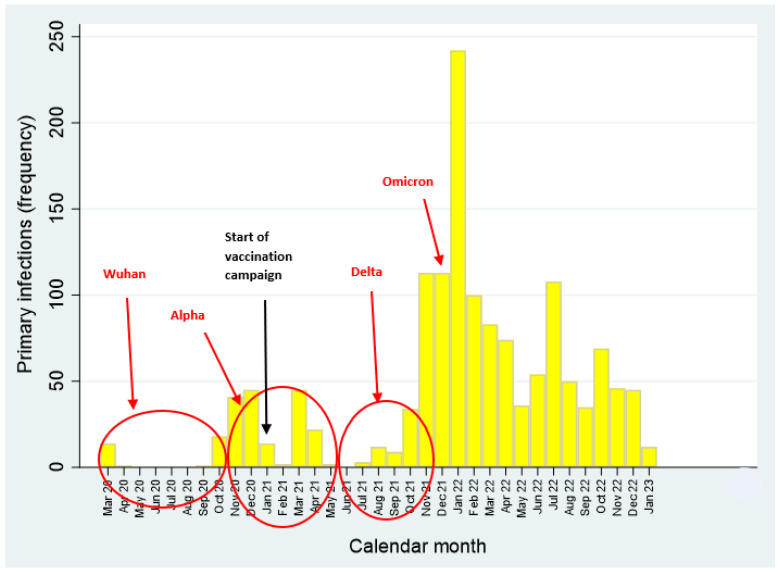
Epidemic curve of primary SARS-CoV-2 infections (1 March 2020–31 January 2023).

**Figure 2:**
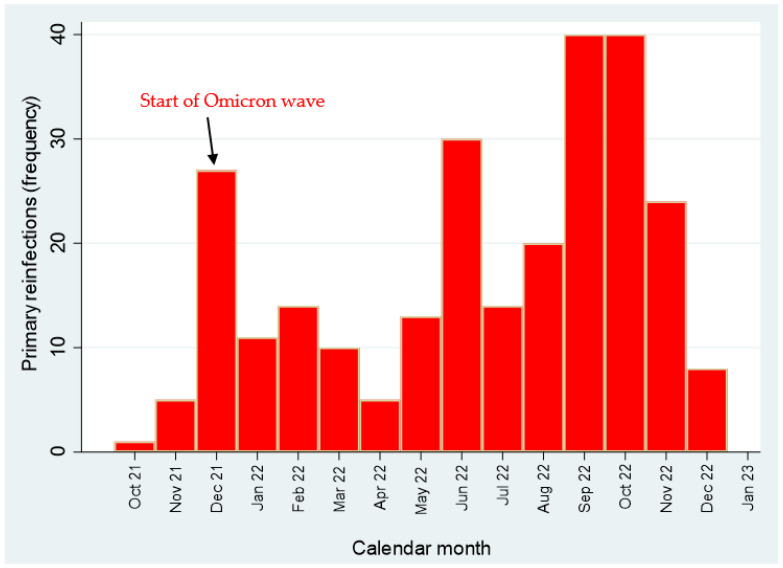
Epidemic curve of primary SARS-CoV-2 reinfections (1 March 2020–31 January 2023).

**Figure 3:**
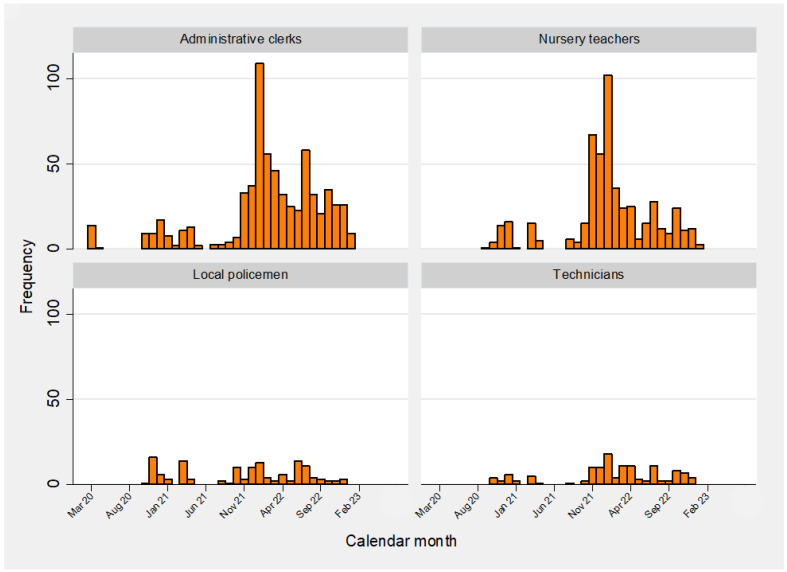
Frequency distribution of primary SARS-CoV-2 infections by calendar month and occupation.

**Figure 4:**
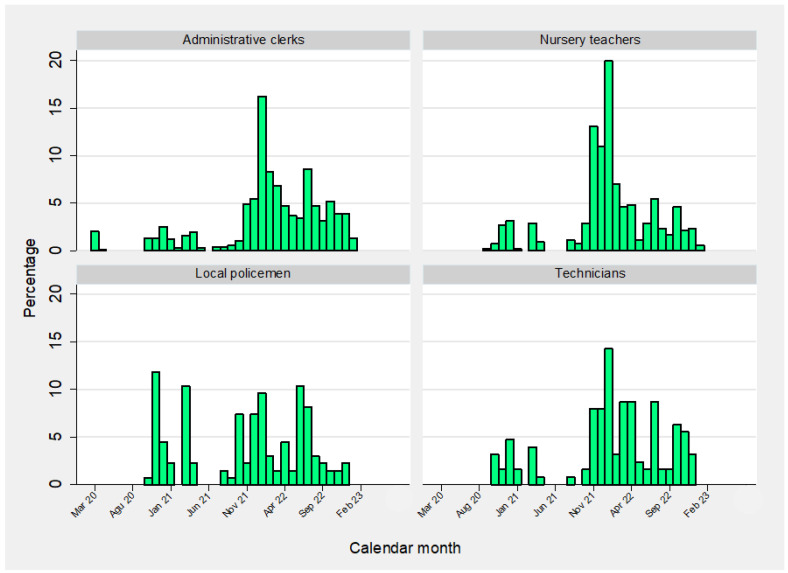
Monthly incidence (percentage) of primary SARS-CoV-2 infections by occupation.

The temporal distribution of primary SARS-CoV-2 infections by calendar month is illustrated in Figure 1 and Figure 2, whereas the epidemic curve of primary COVID-19 infections by occupation is displayed in Figure 3 (frequency distribution) and Figure 4 (percentages).

Table 2 shows the distribution of SARS-CoV-2 infections (primary infection, primary reinfection, second reinfection or third reinfection) by pandemic waves, corresponding to the predominant transmission periods of the main SARS-CoV-2 variants as follows:

- **Wuhan**: 1 March 2020–31 October 2020;
- **Alpha**: 1 November 2021–31 May 2021;
- **Delta**: 1 June 2021–30 November 2021;
- **Omicron**: 1 December 2021–31 January 2023.

As mentioned above, during the entire study period (1 March 2020–31 January 2023) 1444 primary SARS-CoV-2 infections were recorded. Of these, 35 cases were notified during the Wuhan wave, 171 during the Alpha wave and 171 during the Delta wave. The vast majority of primary COVID-19 infections increased from December 2021 onwards (during omicron transmission period), peaking in January 2022. (Table 2). As can be noticed, the Omicron transmission period (1 December 2021–31 January 2023) was featured by the highest transmissibility of SARS-CoV-2, with 1067 workers infected at least once, and 262 at least twice (Table 2). The totality of SARS-CoV-2 reinfections (N=262) occurred during the Omicron transmission period.

Moreover, the mean time since last vaccination received through SARS-CoV-2 infection increased during the course of the pandemic and with number of doses of COVID-19 vaccine received (Table 2).

Table 3 shows the cumulative vaccine uptake by type of COVID-19 vaccine and number of doses.

The national vaccination campaign against COVID-19 started on 27 December 2020 in Italy, initially prioritizing vulnerable individuals and HCWs. For civil servants of Trieste city council, the first vaccine doses started to be delivered on 3 January 2021, second doses started on 24 January 2021, third doses on 24 September 2021, and fourth doses started to be administered on 2 April 2022.

As can be seen from Table 3, a total of 5129 vaccine doses were administered during the entire study period, including 1846 first doses, 1726 second doses, 1411 third doses, 141 fourth doses and 5 fifth doses.

By the end of the study period (31 January 2023), 78% (=1805/2314) of workers were immunized with at least two doses of COVID-19 vaccine versus 19.7% (=457/2314) remaining fully unvaccinated. The majority of workers (55.0% = 1272/2314) were immunized with three doses, 16.9% (=390/2314) with only two, 2.2% (=52/2314) received just one dose, and 6.0% (=138/2314) workers were vaccinated with four doses.

The most used vaccine for every dose was Comirnaty (Pfizer BioNTech, New York City, USA), accounting for 52.1% of all doses administered overall, followed by Spikevax (Moderna, Cambridge, Massachussets, USA) (27.0%). Spikevax was used in 20% of second doses and 46.9% of third doses. Vaxzevria (Oxford–Astrazeneca, Cambridge, UK) ranked third (18.5%) and was mainly used for first and second doses (28.0% and 25.1%, respectively). Jannsen (Johnson & Johnson, New Brunswick, New Jersey, USA) was only used as first dose in 40 workers, accounting for 0.8% of total COVID-19 vaccinations (Table 3).

Supplementary Table S1 and Figure S1 provide a detailed distribution of COVID-19 vaccine uptake by number of doses, type of COVID-19 vaccine received and calendar month. 

Supplementary Table S2 shows the incremental and cumulative number of COVID-19 immunizations by calendar month and vaccine type. Of note, the highest number of vaccinations (N = 951) was delivered in May 2021, with 521 first doses (of which 303 were Comirnaty and 162 were Spikevax) and 430 second doses (of which 324 immunizations were with Vaxzevria and 99 were with Comirnaty). The administration of second doses increased during June 2021, when 667 vaccinations were delivered—478 second doses (of which 277 immunizations were with Vaxzevria and 77 were with Comirnaty) and 189 first doses (of which 160 immunizations were with Comirnaty).

In December 2021, at the beginning of the Omicron wave, 744 vaccinations were administered, of which 660 were third doses (413 Comirnaty versus 247 Spikevax). The peak of third doses continued in January 2022, when 492 immunizations were delivered, of which 404 were third doses (376 Spikevax versus 28 Comirnaty) (Supplementary File S3).

Table 4 displays the distribution of primary SARS-CoV-2 infections, person-days at risk (P-d) × 10,000 and crude incidence rate (95%CI) × 10,000 by explanatory factors. As can be seen, during the entire study period (1 March 2020–31 January 2023), the crude incidence rate of SARS-CoV-2 infections was slightly higher among females (7.74 × 10,000 P-d) compared to males (6.73 × 10,000 P-d). The 40–49 years age group exhibited the highest incidence rate of COVID-19 (8.14 × 10,000 P-d), whereas the other age groups had similar incidence rates, ranging from 7.11 to 7.33 × 10,000 P-d. In terms of occupation, the incidence rates were higher in local police officers (8.92 × 10,000 P-d) and nursery teachers (8.18 × 10,000 P-d), whereas they were lower for administrative clerks (6.95 × 10,000 P-d) and technicians (6.34 × 10,000 P-d). The crude incidence rates of primary infections progressively increased over time during the course of the pandemic in all occupations, but in police officers they were substantially higher already during the Alpha wave (11.54 × 10,000 P-d), decreased during the Delta wave (4.47 × 10,000 P-d) and rose again with Omicron (11.86 × 10,000 P-d) (Table 4).

As can be seen from Table 4, the incidence rate of COVID-19 across the entire study period progressively reduced from 15.47 × 10,000 P-d for one vaccine dose to 10.30 × 10,000 P-d for two doses, 6.10 × 10,000 P-d for three doses and 4.41 × 10,000 P-d for four doses. The crude incidence rate of COVID-19 was remarkably lower with two vaccine doses during Alpha (0.10 × 10,000 P-d) or Delta (3.10 × 10,000 P-d) and four doses during Omicron (2.98 × 10,000 P-d). The crude incidence rates of primary infections were slightly lower for homologous (12.6 × 10,000 P-d) versus heterologous (14.4 × 10,000 P-d) triple vaccination (Table 4).

Table 5 shows the crude incidence rates of primary SARS-CoV-2 reinfections estimated since the date of primary COVID-19 infection by explanatory factors. As can be seen, apart from slightly increased rates for the 40–49 years age group and nursery teachers, the risk of reinfections decreased with number of doses of COVID-19 vaccine, from 9.94 × 10,000 P-d among unvaccinated to 6.69 × 10,000 P-d for those immunized with one dose, 5.01 × 10,000 for those immunized with two doses, 3.43 × 10,000 P-d for those immunized with three doses and 1.64 × 10,000 P-d for those immunized with four doses. The crude incidence rates of primary reinfections were again lower for homologous (12.4 × 10,000 P-d) versus heterologous (15.0 × 10,000 P-d) triple vaccination (Table 5).

Table 6 shows the results of multivariable Cox proportional regression analysis investigating the risk of primary SARS-CoV-2 infections and reinfections adjusted for sex, age, occupation and number of vaccine doses received before infection (14+ days since first dose or 7+ days since 2+ doses). As can be seen, the risk of primary SARS-CoV-2 infections over the entire study period slightly increased in females (aHR = 1.28; 95%CI: 1.12; 1.45) or workers aged 40–49 years (aHR = 1.65; 95%CI: 1.01; 2.71). In terms of occupation, local police officers (aHR = 1.27; 95%CI: 1.13; 1.43) and nursery teachers (aHR = 1.82; 95%CI: 1.50; 2.22) were more likely to be infected compared to administrative clerks (reference category). Moreover, the risk of primary COVID-19 infections was higher in those immunized with just one dose (aHR = 2.12; 95%CI: 1.55; 2.90), whereas it was reduced in workers immunized with three (aHR = 0.42; 95%CI: 0.36; 0.47) or four doses (aHR = 0.30; 95%CI: 0.23; 0.40), for a VE ( = 1-aHR) of 58% and 70%, respectively.

Sub-analysis by main pandemic waves confirmed the higher risk of infection among nursery teachers and especially local police officers. However, whilst in police officers the risk of COVID-19 was already higher during the Alpha wave (aHR = 6.82; 95%CI: 4.48; 10.40), decreasing with Omicron (aHR = 1.43; 95%CI: 1.11; 1.84), for nursery teachers the infection risk increased during the Delta wave (aHR = 2.42; 95%CI: 1.70; 3.44), reducing with Omicron (aHR = 1.23; 95%CI: 1.07; 1.40) (Table 6).

Furthermore, the risk of primary infections was significantly lower with one (aHR = 0.25; 95%CI: 0.16; 0.39) or two doses (aHR = 0.01; 95%CI: 0.00; 0.08) during the Alpha wave, for a VE of 75% and 99%, respectively. The risk of primary infection slightly increased during the Delta period with one (aHR = 0.41; 95%CI: 0.17; 0.93) or two vaccine doses (aHR = 0.30; 95%CI: 0.22; 041), for a VE reducing to 59% and 70%, respectively. During the Omicron wave the risk of primary COVID-19 infections was significantly lower in individuals vaccinated with three (aHR = 0.42; 95%CI: 0.36; 0.49) or four doses (aHR = 0.09; 95%CI: 0.54; 0.16), for a VE of 58% and 91%, respectively (Table 6).

The above pattern was consistently observed for the risk of primary COVID-19 reinfections, where vaccination status was the only determinant. In particular, workers immunized with one (aHR = 0.47; 95%CI: 0.27; 0.82), two (aHR = 0.42; 95%CI: 0.30; 0.58), three (aHR = 0.32; 95%CI: 0.24; 0.44) or four doses (HR = 0.14; 95%CI: 0.05; 0.46) were less likely to be reinfected by SARS-CoV-2 compared to unvaccinated colleagues, for a VE of 53%, 58%, 68% and 86%, respectively (Table 6).

Finally, sub-group analysis limited to workers immunized with 3+ doses (from 23 September 2021 onward) did not show any difference in VE against primary SARS-CoV-2 infections or reinfections by heterologous versus homologous triple vaccination (Table 6).

## 4. Discussion

### 4.1. Main Findings

From 1 March 2020 to 31 January 2023, 1444 (62.4%) employees of Trieste city council tested positive for SARS-CoV-2 at least once and 262 (18.1%) at least twice. Only 20 workers were admitted to hospital for severe disease, hence 99.9% of SARS-CoV-2 infections were mild to moderate. Only one worker, previously immunized with triple vaccination, died. By the end of the study period (31 January 2023), 25,458,763 cumulative COVID-19 cases had been reported in Italy since the start of the pandemic [11].

The vast majority of primary infections (69.3%) occurred during the predominant Omicron transmission period (1 December 2021–31 January 2023), when almost the totality of reinfections (first, second or third) were observed.

The mean time since the last vaccination received throughout primary SARS-CoV-2 infection increased over the course of the pandemic and with number of doses of COVID-19 vaccine received.

By the end of the study, 78% of workers had been immunized with at least two doses of COVID-19 vaccine compared to 20% who remained fully unvaccinated. The most used vaccine type for any dose was Comirnaty, accounting for 52.3% of all immunizations, followed by Spikevax (27.0%), Vaxzevria (18.6%) and Jannsen (0.8%). The peak of vaccinations occurred in May 2021 (N = 951), when second doses started to be administered, followed by December 2021 (N = 744), in coincidence with the massive delivery of third doses.

At multivariable Cox regression analysis, the risk of primary SARS-CoV-2 infections during the entire study period increased in females, workers aged 40–49 years, local police officers and nursery teachers. However, whilst police officers were already at higher risk during the Alpha wave, for nursery teachers, the risk increased during the Delta wave, reducing with Omicron in both latter occupational categories

The main (protective) determinant of SARS-CoV-2 infection during the entire study period was COVID-19 vaccination status, for a VE of 58% with three doses versus 70% with four. Sub-group analysis by pandemic wave revealed a significantly reduced risk of primary infection with first and second vaccine doses during the predominant Alpha and Delta transmission periods, for a VE of 75% and 99% during Alpha, respectively, slightly reducing to 59% and 70% during the Delta wave. With Omicron, the risk of SARS-CoV-2 infections was significantly lower with three or four doses, for a VE of 58% and 91%, respectively. Moreover, the risk of SARS-CoV-2 reinfections also consistently diminished with number of doses of vaccine received, for a VE of 53% with one, 58% for with two, 69% with three and 86% with four doses. 

Finally, no difference in VE was observed with heterologous versus homologous triple vaccination against both primary SARS-CoV-2 infections or reinfections.

### 4.2. Interpretation of Findings

#### 4.2.1. Occupational Risk

COVID-19 infections and mortality reportedly varied over time and by occupation during the pandemic [4,5,6,12,13,14,15,16,17], being influenced by a number of factors, including vaccine uptake, indoor versus outdoor work environment, poor ventilation, exposure to contaminated surfaces, number of people in the worksite, social distancing, efficiency of contract tracing and observation of non-pharmaceutical risk reduction measures [5,6,18,19].

From 31 May 2020 onward, with the end of the country lockdown in Italy, Trieste city council implemented non-pharmaceutical measures to prevent the spread of SARS-CoV-2 in occupations involving interpersonal contact, especially between civil servants and the public. These interventions included mandatory use of face masks indoor until the end of April 2022, social distancing, systematic fomite disinfection, recommendation of hand washing, frequent ventilation of indoor spaces and contact tracing. Smart working was allowed for vulnerable employees. However, these interventions have never been formally assessed using checklists such as the ILO-WHO HealthWISE tool for healthcare premises, for instance [20]. Furthermore, pharmaceutical risk reduction measures such as intranasal administration of natural, harmless and inexpensive biocidal compounds such as seawater were not recommended as pre- or post-exposure prophylaxis [21,22,23,24,25].

Compared to administrative clerks—who were allowed to work remotely during country lockdown and, to some extent, also afterward—the risk of primary COVID-19 infections was significantly higher in nursery teachers and local police officers in the present study. However, whilst police officers were already at higher risk during the Alpha transmission period, for nursery teachers, the biological risk increased during the Delta wave, reducing with Omicron in both occupational groups. The latter figures likely reflect work suspensions during country lock-down and allowance to work remotely for non-essential occupations during the early pandemic phases.

Occupational exposure to SARS-CoV-2 was reportedly higher in the early phase of the pandemic—particularly for essential workers such as HCWs, protective services/police officers, education workers and transportation workers [25], while non-essential occupations were suspended [1,15]. Police officers were exposed to a higher biological risk due to close contact with members of the public and their task of law enforcement to contain the spread of SARS-CoV-2 [26]. A study conducted in Germany during the first pandemic wave reported a higher risk of infection in essential workers—healthcare, logistics, transportation, police, jurisdiction and public administration—and highly skilled professions [27]. According to a study estimating exposure to SARS-CoV-2 based on pre-lockdown working conditions in France, following HCWs, higher levels of exposure were found in army/police officers, firefighters, hairdressers, teachers, cultural/sports professionals and some manual workers [28]. Likewise, in a Brazilian matched case–control study contrasting 1724 cases versus 1741 controls tested by RT-PCR from April 2020 to May 2021, the adjusted risk of COVID-19 was significantly higher in police and protective services (OR = 2.21; 95%: 1.27–3.84), HCWs (OR 1.90; 95%CI 1.34–2.68) and in food, retail and production activities (OR = 1.88; 95%CI = 1.14–3.11), after removing the effect of COVID-19 vaccination and other factors [29].

With the resumption of non-essential activities post-lockdown in Italy, the biological risk increased in occupations with higher levels of social interactions, such as education and transportation [6,30]. In a study on 3241 school workers conducted in Qatar from February 2020 to February 2022, COVID-19 infections progressively increased over time, being lower during the Wuhan wave (N = 113) and peaking during Omicron time (N = 386) when service personnel (HR = 3,5; 1.8–6.9) were at significantly higher biological risk than teachers [31]. A study from Salt Lake City (UTAH, USA) on children and staff members highlighted the critical importance of implementing infection prevention and control measures in nurseries [32]. Whilst typically developing mild or asymptomatic COVID-19, children contribute to SARS-CoV-2 transmission in adults, especially considering their low compliance with health protection measures and that face masks are not recommended for children under 2 years of age [32]. 

#### 4.2.2. Vaccine Effectiveness

COVID-19 vaccination was the main (protective) determinant against the biological risk in the present study, regardless of the pandemic wave. Although designed to prevent morbidity and mortality [33,34,35], COVID-19 vaccinations also proved effective in reducing the risk of asymptomatic SARS-CoV-2 infections [3,5] and the impact of post-COVID-19 sequelae [36] in post-authorization observational studies. Regardless of the vaccine type (inactivated or m-RNA), higher number of doses of COVID-19 vaccines increased protection from SARS-CoV-2 infections among HCWs in various studies conducted during the Omicron and Delta waves [3,5,37,38,39,40]. On the one hand, the present study confirmed the protective effect of COVID-19 vaccination against symptomatic SARS-CoV-2 infections; on the other hand, it highlighted not only the importance of continuous booster doses to keep up the humoral immunity over time, but also the importance of updated vaccine formulations to prevent and control the spread of a highly mutable virus. Moreover, the protective effect of the first or second dose against reinfections confirmed the efficacy of hybrid immunity during the predominant Omicron transmission period [41]

COVID-19 vaccine uptake reportedly differs by occupation [18,42,43,44]. According to a UK Virus Watch study, workers in transportation, trade, sales and service had the lowest COVID-19 vaccine uptake [43], whereas 2021 UK Census data reported high vaccine coverage in office-based and professional workers and low uptake among individuals working in elementary occupations [44]. 

By the end of the present study, 20% of workers of Trieste city council remained unvaccinated, and some of them may have been suspended from work by Italian law from January 2021 to April 2022, unless they were allowed to work remotely. Italy enforced the strongest vaccination policy within the European Union, making COVID-19 vaccines mandatory for all HCWs and any other occupation entailing social interaction with colleagues and the public, including civil servants. Other countries adopted a prioritized vaccination policy for occupational categories at higher biological risk [18].

In a study conducted in England between 1 December 2020 and 11 May 2022, the risk of SARS-CoV-2 infection was highest in educational staff, social caregivers and police/protective services, categories with high coverage of triple vaccination [18].

According to epidemiological evidence from Belgium in the Autumn of 2020, risk reduction measures for close-proximity occupations were sufficient to prevent and control COVID-19 in circumstances of low viral circulation, progressively reducing with increasing circulation of SARS-CoV-2 [9]. With Omicron, a highly transmissible variant, non-pharmaceutical risk reduction measures were more effective than vaccination to prevent asymptomatic COVID-19 in a highly immunized population such as HCWs, who largely contracted the infection outside healthcare premises, where health protection measures were relaxed [4]. 

However, with the spread of Omicron, COVID-19 mortality and morbidity considerably reduced and concentrated on high-risk patients. Identifying groups with low vaccine uptake can inform secondary preventative strategies focusing on the immunization of risky sub-groups, considering the low vaccine uptake reported for some high-risk populations [45].

#### 4.2.3. Heterologous Triple Vaccination

In line with a previous investigation on university staff of Trieste [6], no significant difference in VE of heterologous versus homologous triple vaccination was observed against primary infections or reinfections in the present study. Although, Cox regression model for reinfections comparing heterologous versus homologous regimen was fitted only on 88 complete observations, hence limited statistical power shall not be neglected.

Heterologous COVID-19 immunization drew international attention for severe side effects such as thrombotic events with thrombocytopenia following administration of DNA-vector vaccines (Vaxzevria or Jannsen) [46,47,48,49]. Vaccination protocols were then amended to also offer Comirnaty as a second dose to individuals who had received Vaxzevria as the primary dose [50]. Heterologous vaccination with either Vaxzevria or Comirnaty as prime or second doses showed robust immunogenicity in animal and human studies [50,51,52]. In a randomized multicenter clinical trial conducted in the UK between February 11 and February 26, 2021 on 830 patients, cellular and humoral responses of two heterologous vaccine prime-boost schedules (Vaxzevria/Comirnaty or Comirnaty/Vaxzevria) at 28 days after the boost dose were not lower than the homologous schedule (Vaxzevra/Vaxzevria) [53]. By contrast, a study on 13,489 HCWs at the University Hospital of Lyon (France), conducted from 15 December 2021 to 21 March 2022, showed that heterologous immunizations were more protective against SARS-CoV-2 infection than homologous regimen, but this was no longer the case after the triple dose [54]. Antibody titers and neutralizing capacity were in fact higher after the third compared to the second dose in the homologous regimen, but not in the heterologous group [54]. Nevertheless, according to a systematic review and meta-analysis, VE of two doses of adenoviral vaccine plus one dose of m-RNA vaccine was comparable to three doses of m-RNA vaccines [55]. 

Heterologous immunization with a third dose seems effective with inactivated vaccines. In two single-center, randomized, controlled, observer-blinded trials conducted in Lianshui County, Jiangsu Province (China), mixed vaccinations of the first two doses of inactivated vaccine (CoronaVac) followed by a third dose of adenoviral vectored COVID-19 vaccine (Convidecia) was persistently more immunogenic at 6 months then three doses of CoronaVac [56].

### 4.3. Strengths and Weaknesses

The present study investigated COVID-19 risk over a long period of time (35 months), encompassing the entire pandemic duration in a relatively large sample of civil servants of Trieste city council, an occupational category largely neglected by studies on COVID-19 surveillance. 

Data used in this study relied on routine healthcare records of SARS-CoV-2 infections, date of any COVID-19 vaccination dose and hospital admission, enabling to not only assess the risk of primary infections and reinfections, but also VE and hybrid immunity. 

Moreover, whilst HCWs were all immunized with m-RNA vaccines in Italy, civil servants were also offered adenoviral vector vaccines (Janssen and especially Vaxzevria) for the first two doses, thereby allowing to compare the efficacy of heterologous versus homologous triple vaccination.

Some occupational categories, especially administrative clerks and technicians, were allowed to work remotely if vulnerable, hence, their biological risk was lower. By contrast, police officers were in service during the entire pandemic and nursery teachers were allowed to work remotely only in the initial part of 2020.

As already mentioned, testing on demand applied to civil servants inevitably missed a large proportion of asymptomatic SARS-CoV-2 infections.

Lastly, information on residual confounders potentially influencing the risk of COVID-19, such as pre-existing comorbidities, was not available.

## 5. Conclusions

COVID-19 risk was higher in local police officers and nursery teachers, due to the intrinsic biological risk associated with social interactions involving their professional activites. However, the stronger determinant of infection was COVID-19 vaccination status, with a risk of primary infections over the entire study period decreasing with three or four vaccine doses, for a VE of 58% and 70%, respectively. The protective effect of COVID-19 vaccination against primary infections was consistently observed across all main pandemic waves, for a VE of 75% for the first dose and 99% for the second dose during Alpha wave, slightly reducing to 59% and 70%, respectively, during the Delta transmission period. With Omicron, the risk of SARS-CoV-2 infection diminished significantly in individuals immunized with three or four doses, for a VE of 58% and 91%, respectively.

Finally, VE against primary reinfections was 53% for one dose, 58% for two doses, 68% with three doses and 86% with three doses, confirming the efficacy of hybrid immunity for one or two doses during the Omicron time.

No difference in VE was observed in relation to heterologous versus homologous triple vaccination, both for primary infections and reinfections. 

Since city council civil servants were swab tested on demand or for contact tracing, COVID-19 risk and VE largely refer to symptomatic SARS-CoV-2 infections.

On the one hand, the present study confirmed the protective effect of COVID-19 vaccination against symptomatic SARS-CoV-2 infections; on the other hand, it highlighted not only the importance of continuous booster doses to keep up the humoral immunity over time but also the importance of updated vaccine formulations to prevent and control the spread of a highly mutable virus.

The efficacy of risk reduction measures in any COVID-19 workplace should be evaluated by validated tools in the future.

## Figures and Tables

**Table 1:** Distribution of primary COVID-19 infections and reinfections by explanatory factors. Number (N), row percentage (%) and *p*-value. IQR = interquartile range.

TERMS	STRATA	TOTAL	Primary Infections		*p*	Primary Reinfections		*p*
			No	Yes		No	Yes	
**Total**		2314 (100)	870 (37.6)	1444 (62.4)		1182 (81.9)	262 (18.1)	
**Sex**	**Females**	1629 (70.4)	577 (35.4)	1052 (64.6)	<0.001 *	325 (82.9)	67 (17.1)	0.527 *
	**Males**	685 (29.6)	293 (42.8)	392 (57.2)		857 (81.5)	195 (18.5)	
**Age** (years)	**Median (IQR)**	53 (46; 58)	54 (47; 59)	53 (46; 58)	0.026 **	53 (46; 58)	51 (44; 57)	0.014 **
	**23–29**	28 (1.2)	11 (39.3)	17 (60.7)	0.215 *	14 (82.4)	3 (17.7)	0.102 *
	**30–39**	222 (9.6)	85 (38.3)	137 (61.7)		113 (82.5)	24 (17.5)	
	**40–49**	594 (25.7)	199 (33.5)	395 (66.5)		306 (77.5)	89 (22.5)	
	**50–59**	1026 (44.3)	400 (39.0)	626 (61.0)		520 (83.1)	106 (16.9)	
	**60+**	444 (19.2)	175 (39.4)	269 (60.6)		229 (85.1)	40 (14.9)	
**Occupation**	**Clerks**	1123 (48.5)	451 (40.2)	672 (59.8)	<0.001 *	567 (84.4)	105 (15.6)	0.003 *
	**Nursery teachers**	764 (33.0)	253 (33.1)	511 (66.9)		399 (78.1)	112 (21.9)	
	**Police officers**	198 (8.6)	63 (31.8)	135 (68.2)		104 (77.0)	31 (23.0)	
	**Technicians**	229 (9.9)	103 (45.0)	126 (55.0)		112 (88.9)	14 (11.1)	

* Chi square test; ** non-parametric Mann–Whitney test.

**Table 2:** Distribution of primary SARS-CoV-2 infections and reinfections by pandemic wave-corresponding to the predominant transmission periods of main SARS-CoV-2 variants (Wuhan, Alpha, Delta, Omicron)- and vaccination status. Numbers (N), column percentage (%) and mean (M) number of days ± standard deviation (SD) since last vaccination dose to COVID-19 event.

PANDEMIC WAVE			Primary Infections (N = 1444)	Reinfections (N = 262)		
				First	Second	Third
**Wuhan (1 Mar 2020–31 Oct 2020)**			**35 (2.4)**	**0**	**0**	**0**
**Alpha** **(1 Nov 2020–** **31 May 2021)**	**Total**	**N (%)**	**171 (11.8)**	**0**	**0**	**0**
	**Unvaccinated or** **infected before 1st dose**	**N (%)**	48 (3.3)			
	**Infected between** **1st–2nd dose**	**N (%)**	18 (1.2)			
		**M ± SD (days)**	18.4 ± 11.5			
	**Infected between** **2nd–3rd dose**	**N (%)**	1 (0.1)			
		**M ± SD (days)**	44			
	**Infected after 3rd dose**	**N (%)**	0			
**Delta** **(1 Jun 2021–** **30 Nov 2021)**	**Total**	**N (%)**	**171 (11.8)**	**0**	**0**	**0**
	**Unvaccinated or** **infected before 1st dose**	**N (%)**	83 (5.7)			
	**Infected between** **1st–2nd dose**	**N (%)**	5 (0.3)			
		**M ± SD (days)**	83.6 ± 72.6			
	**Infected between** **2nd–3rd dose**	**N (%)**	80 (5.5)			
		**M ± SD (days)**	219 ± 57.2			
	**Infected after 3rd dose**	**N (%)**	0			
**Omicron** **(1 Dec 2021–** **31 Jan 2023)**	**Total**	**N (%)**	**1067 (73.9)**	**262 (100)**	**9 (100)**	**2 (100)**
	**Unvaccinated or** **infected before 1st dose**	**N (%)**	237 (16.4)	114 (43.5)	5 (50.0)	1 (50.0)
	**Infected between** **1st–2nd dose**	**N (%)**	23 (1.6)	30 (11.5)	2 (0.2)	0
		**M ± SD (days)**	199.7 ± 195.1	338.2 ± 153.1	389 ± 72.8	0
	**Infected between** **2nd–3rd dose**	**N (%)**	224 (15.5)	59 (22.5)	1 (0.1)	0
		**M ± SD (days)**	263.6 ± 99.6	447.1 ± 126.7	321	0
	**Infected between** **3rd–4rd dose**	**N (%)**	575 (39.8)	70 (26.7)	1 (0.1)	1 (50)
		**M ± SD (days)**	423.7 ± 113.9	516.9 ± 112.4	444	600

**Table 3:** Cumulative COVID-19 vaccine uptake by dose number and vaccine type. Number (N), percentage (%). M = missing values.

Dose	Comirnaty	Spikevax	Vaxzevria	Janssen	Comirnaty Bivalent 1 *	Comirnaty bivalent 2 **	Total
**1** (M:2)	918 (49.7)	370 (20.0)	518 (28.0)	40 (2.2)			**1846 (36.0)**
**2** (M:2)	943 (54.6)	349 (20.2)	434 (25.1)				**1726 (33.67)**
**3** (M:2)	740 (52.4)	662 (46.9)			1 (0.1)	8 (0.6)	**1411 (27.5)**
**4** (M:2)	75 (53.2)	5 (3.5)			6 (4.3)	55 (39.0)	**141 (27.5)**
**5**	1 (20.0)					4 (80.0)	**5 (0.1)**
**Total**	**2677 (52.1)**	**1386 (27)**	**952 (18.5)**	**40 (0.8)**	**7 (0.1)**	**67 (1.3)**	**5129 (100)**

* Wuhan/Omicron BA.1; ** Wuhan/Omicron BA.4.5.

**Table 4:** Crude incidence rates of primary SARS-CoV-2 infections during the entire study period (1 March 2020– 31 January 2023), by main pandemic waves and explanatory factors. Number of primary infections (N), person-days (P-d) at risk × 10,000 and raw incidence (×10,000). Number of infections counted 14+ days since 1st vaccine dose or 7+ days since 2+ doses.

TERMS	STRATA	Entire Period (1 Mar 2020–31 Jan 2023) (N. Infections = 1444)			Wuhan Wave (1 Mar 2020–31 Oct 2020) (N. Infections = 35)			Alpha Wave (1 Nov 2020–31 May 2021) (N. Infections = 171)			Delta Wave (1 Jun 2021–30 Nov 2021) (N. Infections = 171)			Omicron Wave (1 Dec 2021–31 Jan 2023) (N. Infections = 1067)		
		N.	P-d × 10,000	Rate × 10,000 (95%CI)	N.	P-d × 10,000	Rate × 10,000 (95%CI)	N.	P-d × 10,000	Rate × 10,000 (95%CI)	N.	P-d × 10,000	Rate × 10,000 (95%CI)	N.	P-d × 10,000	Rate × 10,000 (95%CI)
**Sex**	**Males**	392	582,718	6.73 (6.09; 7.43)	26	167,571	0.54 (0.28; 1.03)	60	137,904	4.35 (3.38; 5.60)	40	124,402	3.21 (2.36; 4.38)	283	215,002	13.16 (11.72; 14.79)
	**Females**	1052	1,359,400	7.74 (7.28; 8.22)	9	397,571	0.65 (0.45; 0.96)	111	332,613	3.34 (2.77; 4.02)	131	296,510	4.42 (4.73; 5.25)	783	481,569	16.25 (15.16; 17.44)
**Age** (years)	**20–29**	17	23,184	7.33 (4.56; 11.80)	0	6888	0	4	5182	7.72 (2.90; 20.57)	1	5147	1.94 (0.27; 13.79)	12	8885	13.51 (7.67; 23.78)
	**30–39**	137	187,998	7.29 (6.16; 8.62)	2	54,572	0.37 (0.09; 1.47)	17	44,876	3.79 (2.35; 6.09)	18	40.349	4.46 (2.81; 7.08)	100	68,310	14.64 (12.03; 17.81)
	**40–49**	395	485,312	8.14 (7.37; 8.98)	5	145,805	0.34 (0.14; 0.82)	48	120,164	4.00 (3.01; 5.30)	66	207,037	6.17 (4.84; 7.85)	276	174,952	15.78 (14.02; 17.75)
	**50–59**	626	867,059	7.22 (6.68; 7.81)	17	250,703	0.68 (0.42; 1.09)	72	208,926	3.45 (2.74; 4.34)	65	186,705	3.48 (2.73; 4.44)	472	308,714	15.26 (13.94; 16.70)
	**60+**	269	378,565	7.11 (6.31; 8.01)	11	107,360	1.02 (0.57; 185)	30	91.369	3.28 (2.06; 4.70)	21	81,272	2.58 (1.68; 3.96)	207	135,710	15.25 (13.31; 17.48)
**Occupation**	**Administrative**	672	967,390	6.95 (6.44; 7.49)	25	272,507	0.92 (0.62; 1.36)	62	231,777	2.67 (2.09 3.43)	50	204,797	2.44 (1.85; 3.22)	535	340,163	15.73 (14.45; 17.12)
	**Nursery teachers**	511	624,610	8.18 (7.50; 8.92)	5	187,833	0.27 (0.11; 0.64)	51	155,619	3.28 (2.49; 4.31)	92	138,117	6.66 (5.43; 8.17)	363	219,356	16.50 (14.89; 18.29)
	**Police officers**	135	151,335	8.92 (7.54; 10.56)	1	48,689	0.21 (0.03; 1.46)	42	36,407	11.54 (8.53; 15.61)	16	35,772	4.47 (2.74; 7.31)	76	64,079	11.86 (9.47; 14.85)
	**Technicians**	126	198,783	6.34 (5.32; 7.55)	4	56,300	0.71 (0.27; 1.89)	16	46,714	3.43 (2.10; 5.59)	13	41,824	3.11 (1.80; 5.35)	93	72,973	12.74 (10.40; 15.62)
**Vaccine doses before infection** (Number)	**0**	344	348,707	9.87 (8.87; 10.96)	NA	NA	NA	146	225,291	6.48 (5.51; 7.62)	84	116,728	7.20 (5.81; 8.91)	239	125,615	19.03 (16.77; 21.60)
	**1**	46	29,737	15.47 (11.59; 20.65)	NA	NA	NA	22	134,001	1.64 (1.08; 2.49)	6	26,259	2.28 (1.03; 5.09)	16	25,410	6.30 (3.85; 10.28)
	**2**	301	292,128	10.30 (9.20; 11.54)	NA	NA	NA	1	104,762	0.10 (0.01; 0.68)	81	261,147	3.10 (2.49; 3.86)	234	137,537	17.01 (14.97; 19.34)
	**3**	694	1,137,887	6.10 (5.66; 6.57)	NA	NA	NA	NA	NA	NA	0	16,008	NA	565	367,892	15.36 (14.15; 16.68)
	**4**	59	133,659	4.41 (3.42; 5.69)	NA	NA	NA	NA	20.448	NA	0	NA	NA	12	40,217	2.98 (1.69; 5.25)
Triple dose * (M = 781)	**Homologous**	418	331,381	12.6 (11.5; 13.9)	NA	NA	NA	NA	NA	NA	NA	NA	NA	401	294,723	13.6 (12.3; 15.0)
	**Heterologous**	187	129,932	14.4 (12.5; 16.6)	NA	NA	NA	NA	NA	NA	NA	NA	NA	170	120,808	14.1 (12.1; 16.4)

* 1 or 2 doses of Vaxzevria for the first two immunizations followed by a triple dose with m-RNA vaccine (Comirnaty or Spikevax) or first dose by Janssen followed by a second and triple dose with m-RNA vaccine (Comirnaty or Spikevax).

**Table 5:** Crude incidence rates of primary SARS-CoV-2 reinfections during the entire study period (1 March 2020–31 January 2023), by explanatory factors. Number of reinfections (N), person-days (P-d) at risk × 10,000 and raw incidence (×10,000). Number of infections counted 14+ days since 1st vaccine dose or 7+ days since 2+ doses. M = missing values.

TERMS	STRATA	N. Reinfections	P-d at Risk × 10,000	Rate × 10,000 (95%CI)
**Sex**	**Females**	67	135,982	4.93 (3.88; 6.26)
	**Males**	195	345,355	5.65 (4.91; 6.50)
**Age** (years)	**20–29**	3	6296	4.76 (1.54; 14.77)
	**30–39**	24	44,421	5.40 (3.62; 8.06)
	**40–49**	89	130,981	6.79 (5.52; 8.36)
	**50–59**	106	210,303	5.04 (4.17; 6.10)
	**>60**	40	89,335	4.48 (3.28; 6.10)
**Job task**	**Administrative**	105	213,019	4.93 (4.07; 5.97)
	**Nursery teachers**	112	170,290	6.58 (5.47; 7.92)
	**Local police officers**	31	54,010	5.74 (4.04; 8.16)
	**Technicians**	14	44,017	3.18 (1.88; 5.37)
**COVID-19 vaccine doses** (Number)	**0**	114	114,650	9.94 (8.28; 11.95)
	**1**	15	22,422	6.69 (4.03; 11.10)
	**2**	58	115,789	5.01 (3.87; 6.48)
	**3**	72	211,157	3.43 (2.72; 4.32)
	**4**	3	18,338	1.64 (0.53; 5.07)
**Triple dose** (M: 202)	**Homologous**	27	21,831	12.4 (8.5; 18.0)
	**Heterologous**	18	11,387	15.0 (10.0; 25.1)

**Table 6:** Multivariable Cox proportional regression analysis investigating the risk of primary SARS-CoV-2 infections and reinfections over the entire study period (1 March 2020–31 January 2023) by main COVID-19 waves (Wuhan, Alpha, Delta and Omicron) and by heterologous vs. homologous triple vaccination. Adjusted hazard ratios (aHR) with 95% confidence intervals (95%CI). Pseudo-R^2^ = coefficient of determination. Number of infectons counted 14+ days since 1st vaccine dose or 7+ days since 2+ doses. Orange (darke/lighter) highlights mark significantly higher infection risks; green highlights (darker/lighter) mark significantly reduced risks of infection.obs.= complete case (analysis) observations.

TERMS	STRATA	PRIMARY INFECTIONS						PRIMARY REINFECTIONS	
		Entire Study Period (1 March 20– 31 January 23)	Wuhan Wave (1 March 20– 31 October 20)	Alpha Wave (1 November 20– 31 May 21)	Delta Wave (1 June 21– 30 November 21)	Omicron Wave (1 Decmber 21– 31 January 23)	Only Workers Immunized with 3+ Doses * (23 September 21– 31 January 23)	Entire Period (1 March 20– 31 January 23)	Only Workers Immunized with 3+ Doses * (23 September 21– 31 January 23)
		aHR (95%CI) (2314 obs.)	aHR (95%CI) (2314 obs.)	aHR (95%CI) (2261 obs.)	aHR (95%CI) (2.106 obs.)	aHR (95%CI) (1937 obs)	aHR (95%CI) (1217 obs.)	aHR (95%CI) (1388 obs.)	aHR (95%CI) (88 obs.)
**Sex**	**Males**	*reference*	*reference*	*reference*	*reference*	*reference*	*reference*	*reference*	*reference*
	**Females**	1.28 (1.12; 1.45)	1.30 (0.57; 2.94)	1.25 (0.87; 1.78)	1.38 (0.93; 2.05)	1.29 (1.10; 1.49)	1.26 (1.03; 1.56)	1.03 (0.76; 1.42)	0.98 (0.42; 2.33)
**Age** (years)	**20–29**	*reference*	*NA*	*reference*	*reference*	*reference*	*reference*	*reference*	*NA*
	**30–39**	1.40 (0.84; 2.33)	0.60 (0.14; 2.60)	0.50 (0.17; 1.50)	2.14 (0.28; 16.14)	1.35 (0.74; 2.48)	0.58 (0.26; 1.28)	1.08 (0.32; 3.63)	1.35 (0.38; 4.76)
	**40–49**	1.65 (1.01; 2.71)	0.59 (0.22; 1.61)	0.68 (0.24; 1.89)	2.66 (0.37; 19.28)	1.58 (0.88; 2.84)	0.69 (0.32; 1.46)	1.17 (0.37; 3.77)	1.60 (0.78; 3.27)
	**50–59**	1.49 (0.91; 2.43)	Reference	0.58 (0.21; 1.60)	1.69 (0.23; 12.22)	1.54 (0.86; 2.75)	0.57 (0.27; 1.21)	0.91 (0.28; 2.89)	reference
	**60+**	1.44 (0.87; 2.37)	1.46 (0.68; 3.11)	0.61 (0.21; 1.74)	1.36 (0.18; 10.18)	1.51 (0.83; 2.71)	0.55 (0.26; 1.19)	0.85 (0.26; 2.79)	0.88 (0.37; 2.12)
**Occupation**	**Admin. clerks**	*reference*	*reference*	*reference*	*reference*	*reference*	*reference*	*reference*	*reference*
	**Nursery teachers**	1.27 (1.13; 1.43)	0.31 (0.12; 0.82)	1.34 (0.91; 1.96)	2.42 (1.70; 3.44)	1.23 (1.07; 1.40)	0.89 (0.72; 1.09)	1.09 (0.83; 1.44)	1.74 (0.80; 3.79)
	**Local police officers**	1.82 (1.50; 2.22)	0.26 (0.03; 1.99)	6.82 (4.48; 10.40)	2.99 (0.68; 2.38)	1.43 (1.11; 1.84)	1.07 (0.76; 1.49)	1.08 (0.69; 1.68)	0.38 (0.08; 1.73)
	**Technicians**	0.92 (0.76; 1.13)	0.80 (0.26; 2.44)	1.20 (0.68; 2.11)	1.27 (0.68; 2.38)	0.85 (0.68; 1.07)	0.78 (0.55; 1.09)	0.56 (0.32; 1.00)	0.45 (0.10; 2.10)
**N. doses of** **COVID-19** **vaccine**	**0**	*reference*	*reference*	*reference*	*reference*	*reference*		*reference*	
	**1**	2.12 (1.55; 2.90)	NA	0.25 (0.16; 0.39)	0.41 (0.17; 0.93)	1.30 (0.78; 2.17)		0.47 (0.27; 0.82)	
	**2**	0.98 (0.84; 1.15)	NA	0.01 (0.00; 0.08)	0.30 (0.22; 041)	1.07 (0.89; 1.29)		0.42 (0.30; 0.58)	
	**3**	0.42 (0.36; 0.47)	NA	NA	NA	0.42 (0.36; 0.49)		0.32 (0.24; 0.44)	
	**4**	0.30 (0.23; 0.40)	NA	NA	NA	0.09 (0.05; 0.16)		0.14 (0.05; 0.46)	
**Triple** vaccinaton *	**Homologous**	NA	NA	NA	NA	NA	*reference*	NA	*reference*
	**Heterologous**	NA	NA	NA	NA	NA	1.15 (0.94; 1.42)	NA	1.19 (0.57; 2.50)
**Pseudo-R^2^**		0.161	0.277	0.076	0.046	0.014	0.002	0.021	0.049

Mathematical equation to estimate the hazard (h) at time: h(t) = h_0_(t) × exp (β_1_ × sex + β_2_ × age + β_3_ × occupation + β_4_ × vaccination status) or h(t) = h_0_(t) * exp (β_1_ × sex + β_2_ × age + β_3_ × occupation + β_4_ × heterologous vaccination), where h_0_(t) is the baseline hazard at time t for an individual in whom all exposure variables = 0. * First two doses by Vaxzevria followed by third dose with m-RNA vaccine or first dose by Janssen followed by a second and third dose by m-RNA vaccine.

## Data Availability

The data generated and analyzed during the current study are not publicly available since they were purposively collected by the authors for the present study, but they are available from the corresponding author upon reasonable request.

## Supplementary Materials

The following supporting information can be downloaded at: https://www.mdpi.com/article/10.3390/vaccines12030254/s1, Figure S1. Cumulative COVID-19 vaccine uptake, by number of doses of vaccine administered over time. Table S1. Cumulative number of doses of COVID-19 vaccine administered over time in the study population (N = 2314). Table S2. Number of doses of COVID-19 vaccine administered over time, by vaccine type.
