# Supplementary material for: COVID-19 in City Council Civil Servants, 1 March 2020–31 January 2023: Risk of Infection, Reinfection, Vaccine Effectiveness and the Impact of Heterologous Triple Vaccination
Source: Vaccines (Basel). 2024 Feb 28;12(3):254. doi: 10.3390/vaccines12030254 (PMC10974058; doi:10.3390/vaccines12030254)
Supplement: Supplementary file 1 [file vaccines-12-00254-s001.zip › vaccines-2837241-supplementary.pdf]

Supplementary Table S1. Cumulative number of doses of COVID-19 vaccine administered over time in the study population (N=2,314).

DATE	0 doses	1 dose	2 doses	3 doses	4 doses	5 doses
**1 Jan 2021**	2314	0	0	0	0	0
**15 Jan 2021**	2257	57	0	0	0	0
**1 Feb 2021**	2249	52	13	0	0	0
**14 Feb 2021**	2235	19	60	0	0	0
**1 Mar 2021**	2101	149	64	0	0	0
**15 Mar 2021**	1868	365	81	0	0	0
**1 Apr 2021**	1736	494	84	0	0	0
**15 Apr 2021**	1690	539	85	0	0	0
**1 May 2021**	1636	558	120	0	0	0
**15 May 2021**	1486	670	158	0	0	0
**1 Jun 2021**	1113	651	550	0	0	0
**15 Jun 2021**	943	677	694	0	0	0
**1 Jul 2021**	923	362	1029	0	0	0
**15 Jul 2021**	915	213	1186	0	0	0
**1 Aug 2021**	893	157	1264	0	0	0
**15 Aug 2021**	832	202	1280	0	0	0
**1 Sep 2021**	777	224	1313	0	0	0
**15 Sep 2021**	725	230	1359	0	0	0
**1 Oct 2021**	617	251	1445	1	0	0
**15 Oct 2021**	639	256	1418	1	0	0
**1 Nov 2021**	582	221	1505	6	0	0
**15 Nov 2021**	572	213	1516	13	0	0
**1 Dec 2021**	553	192	1469	100	0	0
**15 Dec 2021**	526	174	1169	445	0	0
**1 Jan 2022**	512	149	892	761	0	0
**15 Jan 2022**	498	129	715	972	0	0
**1 Feb 2022**	472	115	562	1165	0	0
**14 Feb 2022**	464	95	531	1224	0	0
**1 Mar 2022**	461	74	528	1251	0	0
**15 Mar 2022**	461	62	517	1274	0	0
**1 Apr 2022**	461	53	495	1305	0	0
**15 Apr 2022**	459	53	472	1329	1	0
**1 May 2022**	459	53	457	1341	4	0
**15 May 2022**	459	53	439	1359	4	0
**1 Jun 2022**	459	53	432	1366	4	0
**15 Jun 2022**	459	53	429	1368	5	0
**1 Jul 2022**	458	53	426	1372	5	0
**15 Jul 2022**	458	52	424	1375	5	0
**1 Aug 2022**	458	52	418	1377	9	0
**15 Aug 2022**	458	52	415	1364	25	0
**1 Sep 2022**	458	52	411	1366	27	0
**15 Sep 2022**	458	51	411	1364	30	0
**1 Oct 2022**	458	51	411	1358	36	0
**15 Oct 2022**	458	51	408	1352	45	0
**1 Nov 2022**	457	52	400	1327	78	0
**15 Nov 2022**	456	52	397	1314	94	1
**1 Dec 2022**	457	52	395	1298	110	2
**15 Dec 2022**	457	52	395	1282	126	2
**1 Jan 2023**	457	52	393	1278	132	2
**15 Jan 2023**	456	52	390	1275	138	3
**31 Jan 2023**	457	52	390	1272	138	5

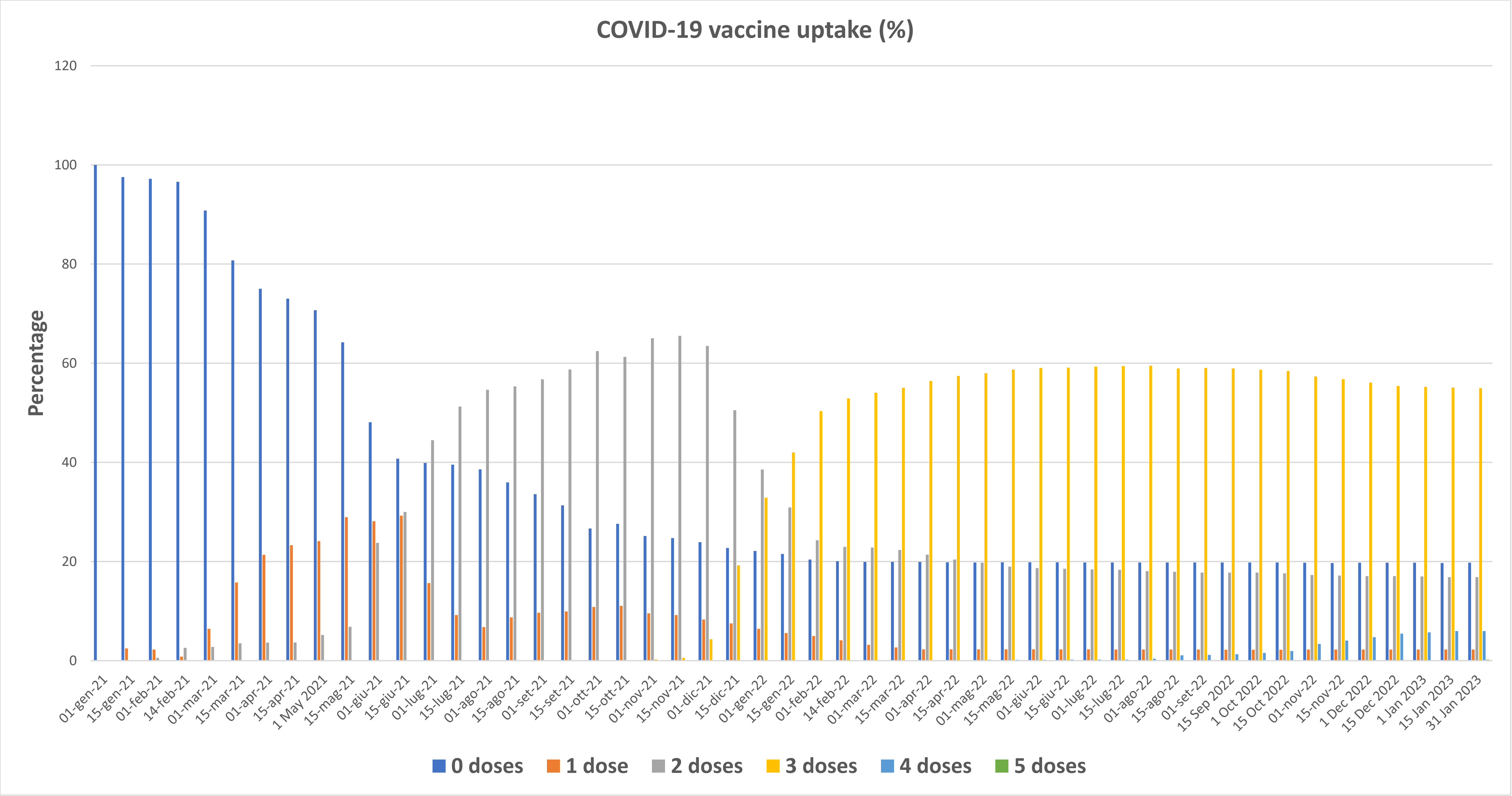

Supplementary Figure S1. Cumulative COVID-19 vaccine uptake, by number of doses of vaccine administered over time

Supplementary Table S2. Number of doses of COVID-19 vaccine administered over time, by vaccine type. M=missing.

Date	VACCINE TYPE	VACCINE DOSES (NUMBER)										TOTAL	
		Dose 1 (M:2)		Dose 2 (M:2)		Dose 3 (M:2)		Dose 4 (M:2)		Dose 5			
		Incremental	Cumulative	Incremental	Cumulative	Incremental	Cumulative	Incremental	Cumulative	Incremental	Cumulative	Incremental	Cumulative
1 January 2021	**Total**	**0**	**0**	**0**	**0**							**0**	**0**
	COMIRNATY											0	0
	SPIKEVAX											0	0
	VAXZEVRIA											0	0
	JANSSEN											0	0
1 February 2021	**Total**	**65**	**65**	**13**	**13**							**78**	**78**
	COMIRNATY	65	65	13	13							78	78
	SPIKEVAX											0	0
	VAXZEVRIA											0	0
	JANSSEN											0	0
1 March 2021	**Total**	**148**	**213**	**51**	**64**							**199**	**277**
	COMIRNATY	16	81	51	64							67	145
	SPIKEVAX											0	0
	VAXZEVRIA	67	132									67	132
	JANSSEN											0	0
1 April 2021	**Total**	**365**	**578**	**27**	**84**							**392**	**662**
	COMIRNATY	19	100	27	84							46	184
	SPIKEVAX											0	0
	VAXZEVRIA	346	478									346	478
	JANSSEN											0	0
1 May 2021	**Total**	**100**	**678**	**36**	**120**							**136**	**798**
	COMIRNATY	85	185	36	120							121	305
	SPIKEVAX	1	1									1	1
	VAXZEVRIA	14	492									14	492
	JANSSEN											0	0
1 June 2021	**Total**	**521**	**1199**	**430**	**550**							**951**	**1749**
	COMIRNATY	303	488	99	219							402	707
	SPIKEVAX	162	163	7	7							169	170
	VAXZEVRIA	26	518	324	324							350	842
	JANSSEN	30	30									30	30

1 July 2021	**Total**	**189**	**1388**	**478**	**1028**							**667**	**2416**
	COMIRNATY	160	648	277	496							437	1144
	SPIKEVAX	19	182	24	131							43	313
	VAXZEVRIA	0	518	77	401							77	919
	JANSSEN	10	40									10	40
1 August 2021	**Total**	**30**	**1418**	**235**	**1263**							**265**	**2681**
	COMIRNATY	26	674	170	666							196	1340
	SPIKEVAX	4	186	37	168							41	354
	VAXZEVRIA	0	518	28	429							28	947
	JANSSEN	0	40									0	40
1 September 2021	**Total**	**116**	**1534**	**49**	**1312**							**165**	**2846**
	COMIRNATY	62	736	31	697							93	1433
	SPIKEVAX	54	240	13	181							67	421
	VAXZEVRIA	0	518	5	434							5	952
	JANSSEN	0	40									0	40
1 October 2021	**Total**	**132**	**1666**	**105**	**1417**	**1**	**1**					**238**	**3084**
	COMIRNATY	132	868	77	774	1	1					210	1643
	SPIKEVAX	0	240	28	209							28	449
	VAXZEVRIA	0	518	0	434							0	952
	JANSSEN	0	40									0	40
1 November 2021	**Total**	**62**	**1728**	**92**	**1509**	**5**	**6**					**159**	**3243**
	COMIRNATY	26	894	92	866	5	6					123	1766
	SPIKEVAX	36	276	0	209							36	485
	VAXZEVRIA	0	518	0	434							0	952
	JANSSEN	0	40									0	40
1 December 2021	**Total**	**28**	**1756**	**53**	**1562**	**94**	**100**					**175**	**3418**
	COMIRNATY	3	897	21	887	84	90					108	1874
	SPIKEVAX	25	301	32	241	10	10					67	552
	VAXZEVRIA	0	518	0	434							0	952
	JANSSEN	0	40									0	40
1 January 2022	**Total**	**40**	**1796**	**44**	**1606**	**660**	**760**					**744**	**4162**
	COMIRNATY	6	903	10	897	413	503					429	2303
	SPIKEVAX	34	335	34	275	247	257					315	867
	VAXZEVRIA	0	518	0	434							0	952

	JANSSEN	0	40									0	40
**1 February 2022**	**Total**	**38**	**1834**	**50**	**1656**	**404**	**1164**					**492**	**4654**
	COMIRNATY	6	909	5	902	28	531					39	2342
	SPIKEVAX	32	367	45	320	376	633					453	1320
	VAXZEVRIA	0	518	0	434							0	952
	JANSSEN	0	40									0	40
**1 March 2022**	**Total**	**10**	**1844**	**49**	**1705**	**86**	**1250**					**145**	**4799**
	COMIRNATY	7	916	22	924	70	601					99	2441
	SPIKEVAX	3	370	27	347	16	649					46	1366
	VAXZEVRIA	0	518	0	434							0	952
	JANSSEN	0	40									0	40
**1 April 2022**	**Total**	**0**	**1844**	**18**	**1723**	**54**	**1304**					**72**	**4871**
	COMIRNATY	0	916	16	940	49	650					65	2506
	SPIKEVAX	0	370	2	349	5	654					7	1373
	VAXZEVRIA	0	518	0	434							0	952
	JANSSEN	0	40									0	40
**1 April 2022**	**Total**	**1**	**1845**	**1**	**1724**	**40**	**1344**					**46**	**4917**
	COMIRNATY	1	917	1	941	39	689					45	2551
	SPIKEVAX	0	370	0	349	1	655					1	1374
	VAXZEVRIA	0	518	0	434							0	952
	JANSSEN	0	40									0	40
**1 May 2022**	**Total**	**0**	**1845**	**0**	**1724**	**25**	**1369**	**4**	**4**			**25**	**4942**
	COMIRNATY	0	917	0	941	21	710	4	4			21	2572
	SPIKEVAX	0	370	0	349	4	659					4	1378
	VAXZEVRIA	0	518	0	434							0	952
	JANSSEN	0	40									0	40
**1 June 2022**	**Total**	**0**	**1845**	**0**	**1724**	**7**	**1376**	**0**	**4**			**8**	**4950**
	COMIRNATY	0	917	0	941	4	714	0	4			5	2577
	SPIKEVAX	0	370	0	349	3	662					3	1381
	VAXZEVRIA	0	518	0	434							0	952
	JANSSEN	0	40									0	40
**1 July 2022**	**Total**	**0**	**1845**	**1**	**1725**	**8**	**1384**	**1**	**5**			**13**	**4963**
	COMIRNATY	0	917	0	942	8	722	1	5			12	2590
	SPIKEVAX	0	370	0	349	0	662					0	1381
	VAXZEVRIA	0	518	0	434							0	952
	JANSSEN	0	40									0	40

**1 August 2022**	**Total**	**0**	**1845**	**0**	**1725**	**0**	**1390**	**4**	**9**			**17**	**4986**
	COMIRNATY	0	917	0	942	0	728	4	9			17	2613
	SPIKEVAX	0	370	0	349	0	662					0	1381
	VAXZEVRIA	0	518	0	434							0	952
	JANSSEN	0	40									0	40
**1 September 2022**	**Total**	**0**	**1845**	**1**	**1726**	**1**	**1391**	**17**	**26**			**11**	**4997**
	COMIRNATY	0	917	1	943	0	728	17	26			4	2617
	SPIKEVAX	0	370	0	349	0	662					0	1381
	VAXZEVRIA	0	518	0	434							0	952
	JANSSEN	0	40									0	40
**1 October 2022**	**Total**	**0**	**1845**	**1**	**1726**	**1**	**1391**	**9**	**35**			**11**	**4997**
	COMIRNATY	0	917	1	943	0	728	3	29			4	2617
	SPIKEVAX	0	370	0	349	0	662					0	1381
	VAXZEVRIA	0	518	0	434							0	952
	JANSSEN	0	40									0	40
	BIVALENT 1					1	1	6	6			7	7
**1 November 2022**	**Total**	**1**	**1846**	**0**	**1726**	**10**	**1401**	**42**	**77**			**53**	**5050**
	COMIRNATY	1	918	0	943	10	738	39	68			50	2669
	SPIKEVAX	0	370	0	349	0	662	3	3			3	1385
	VAXZEVRIA	0	518	0	434							0	952
	JANSSEN	0	40									0	40
	BIVALENT 1					0	1	0	6			0	7
**1 December 2022**	**Total**	**0**	**1846**	**0**	**1726**	**2**	**1408**	**34**	**111**	**2**	**2**	**23**	**5112**
	COMIRNATY	0	918	0	943	0	740	7	75	1	1	7	2677
	SPIKEVAX	0	370	0	349	0	662	1	4			1	1387
	VAXZEVRIA	0	518	0	434							0	953
	JANSSEN	0	40									0	40
	BIVALENT 1					1	1	0	6			0	7
	BIVALENT 2					0	3	26	26	**1**	**1**	26	29
**1 January 2023**	**Total**	**0**	**1846**	**0**	**1726**	**2**	**1408**	**21**	**132**	**0**	**2**	**23**	**5112**
	COMIRNATY	0	918	0	943	0	740	0	75	0	1	0	2677
	SPIKEVAX	0	370	0	349	0	662	1	5			1	1391
	VAXZEVRIA	0	518	0	434							0	957
	JANSSEN	0	40									0	40
	BIVALENT 1					0	1	0	6			0	7
	BIVALENT 2					2	5	20	46	0	1	22	51

	**Total**	**0**	**1846**	**0**	**1726**	**3**	**1411**	**9**	**141**	**3**	**5**	**12**	**5124**
	COMIRNATY	0	918	0	943	0	740	0	75	0	1	0	2677
	SPIKEVAX	0	370	0	349	0	662	0	5			0	1386
**1 February 2023**	VAXZEVRIA	0	518	0	434							0	952
	JANSSEN	0	40									0	40
	BIVALENT 1					0	1	0	6			0	7
	BIVALENT 2					3	8	9	55	3	4	12	67
**TOTAL**			**1846**		**1726**		**1411**		**141**		**5**		**5129**

* Bivalent: WUHAN/OMICRON BA.1.5

** Bivalent= WUHAN/OMICRON BA.4.5
